# Response to chemotherapy could predict the prognosis of esophageal squamous cell carcinoma treated with neoadjuvant docetaxel, cisplatin, and fluorouracil (DCF) followed by surgery: long-term results in a single institute

**DOI:** 10.1007/s10388-024-01062-y

**Published:** 2024-07-10

**Authors:** Yasuyoshi Sato, Kazuhiko Mori, Shinichiro Atsumi, Kei Sakamoto, Shuichiro Oya, Asami Okamoto, Masayuki Urabe, Yoshiyuki Miwa, Shoh Yajima, Koichi Yagi, Sachiyo Nomura, Hiroharu Yamashita, Yasuyuki Seto

**Affiliations:** 1 Department of Gastrointestinal Surgery, Graduate School of Medicine, The University of Tokyo, Tokyo, Japan; 2 Department of Clinical Oncology, The University of Tokyo Hospital, 7-3-1 Hongou, Bunkyo-ku, Tokyo, 113-8655 Japan; 3 Department of Gastroenterological Surgery, Mitsui Memorial Hospital, Tokyo, Japan; 4 Department of Digestive Surgery, Nihon University School of Medicine, Tokyo, Japan

**Keywords:** Esophageal cancer, Esophageal squamous cell carcinoma, Neoadjuvant, DCF, Response to chemotherapy

## Abstract

**Background:**

Preoperative chemotherapy with 5-fluorouracil and cisplatin (FP) followed by surgery has been considered a standard treatment for patients with stage II/III esophageal squamous cell carcinoma (ESCC) based on the results of a phase III trial (JCOG9907) in Japan. Subsequently, the phase III NExT trial (JCOG1109) revealed the survival benefit of the neoadjuvant DCF regimen, which adds docetaxel to FP, and it became a standard treatment. However, the long-term results and prognostic factors of neoadjuvant DCF therapy in the real world are unknown.

**Methods:**

We retrospectively investigated 50 patients with ESCC treated with neoadjuvant DCF therapy from July 2012 to December 2017 at The University of Tokyo Hospital.

**Results:**

Median overall survival (OS) and progression-free survival (PFS) were 32.3 [95% confidence interval (CI) 21.0–NA] and 10.0 months (95% CI 6.3–15.6), respectively. Median OS [not reached (95% CI 31.5–NA) vs. 21.4 months (95% CI 13.5–33.0); p = 0.028] and PFS [83.3 months (95% CI 6.4–NA) vs. 7.4 months (95% CI 6.0–12.8] were significantly longer in patients with an objective response than in non-responders. Of 44 surgical cases, median PFS tended to be longer in pathological lymph node metastasis-negative patients. Conversely, survival did not differ according to cStage (II/III vs. IV) or the average relative dose intensity (ARDI, ≥ 85% vs. < 85%).

**Discussion:**

The response to neoadjuvant DCF therapy could predict patient prognosis. Additionally, pN+ tended to increase the recurrence risk, whereas cStage and ARDI did not influence survival.

## Introduction

Esophageal cancer is an incurable cancer with a high recurrence rate even after early diagnosis and local therapy such as surgery. Esophageal cancer caused 11,619 deaths in Japan in 2019, accounting for 3.1% of all cancer deaths in the country. In recent years, the mortality rate has declined but the morbidity rate has remained on an upward trend. The number of esophageal cancer cases in Japan was 25,438 in 2019, making it the sixth most common cancer among Japanese men [1].

Based on the JCOG9204 study comparing postoperative cisplatin plus 5-FU (FP) to surgery alone [2] and the JCOG9907 study comparing preoperative FP to postoperative chemotherapy [3], preoperative chemotherapy with FP followed by radical surgery has been the standard treatment for resectable cStage IB/II/III esophageal cancer in Japan. However, the 5-year survival rate of 55% in the JCOG9907 trial was suboptimal, and preoperative chemotherapy with more aggressive regimens has been tested.

DCF therapy, which adds docetaxel to FP therapy, has been developed as a neoadjuvant therapy for gastric cancer [4] and head and neck cancer [5, 6]. For esophageal cancer, a phase II trial reported in 2007 enrolled patients with T4 esophageal cancer without distant metastasis who received DCF followed by chemoradiotherapy [7]. In Japan, several phase II trials of preoperative DCF therapy for stage II/III esophageal cancer were conducted. Their response rates ranged 62–72%, and the R0 resection rates ranged 88–95% [8, 9]. Based on these results, we adopted neoadjuvant DCF therapy followed by surgery for patients with locally advanced esophageal cancer starting in January 2013.

Then, JCOG1109 (NExT), a randomized phase III trial comparing preoperative FP with preoperative DCF and chemoradiotherapy with cisplatin (FP-RT) in patients with resectable locally advanced esophageal squamous cell carcinoma (ESCC) was conducted. Median overall survival (OS) was 5.6 years in the FP group [95% confidence interval (CI) 3.9–NE], versus not reached in the DCF group (95% CI 6.7–NE) and 7.0 years in the FP-RT group (95% CI 5.2–NE). Compared with FP, the results demonstrated the superiority of DCF [hazard ratio (HR) 0.68, 95% CI 0.50–0.92, p = 0.006) but not FP-RT (HR 0.68, 95% CI 0.50–0.92, p = 0.006) [10]. Based on these results, on February 3, 2022, the Japanese Esophageal Association Guidelines Committee issued a preliminary report designating preoperative DCF therapy as the new standard of care for stage II and III esophageal cancer. However, the long-term results and prognostic factors of neoadjuvant DCF therapy followed by surgery in the real world are unknown.

## Patients and methods

We retrospectively analyzed data prospectively collected from patients with ESCC who received DCF as preoperative chemotherapy from July 2012 to December 2017 at The University of Tokyo Hospital. The database recorded the following patient characteristics: age, sex, histological diagnosis, primary tumor location, and TNM classification according to the International Union Against Cancer (UICC) 7th Edition [11].

The DCF regimen consisted of IV docetaxel (70 mg/m^2^) on day 1, IV cisplatin (60–70 mg/m^2^) on day 1, and a continuous infusion of fluorouracil (700 mg/m^2^) on days 1–5. This regimen was repeated every 4 weeks for two cycles unless unacceptable adverse events occurred. Dose reductions were permitted at the physician’s discretion. Dosing was adjusted or discontinued depending on the condition of each patient. In the later NExT trial, preoperative DCF treatment was performed 3 courses every 3 weeks [10], however, our DCF regimen had been performed in 2 courses by simply adding docetaxel to the FP regimen of the JCOG9907 trial [3] and done every 4 weeks concerned about adverse events such as bone marrow suppression until 2017.

OS and progression-free survival (PFS) were estimated using the Kaplan–Meier method and log-rank test. Data were censored on September 30, 2023. Patients who were lost to follow-up were censored at the date of last contact or follow-up. OS was calculated from the date of DCF therapy initiation to the date of death from any cause. Patients who were alive on September 30, 2023 were censored for OS analysis. PFS was calculated from the date of DCF therapy initiation to the date of disease progression, recurrence, or death from any cause.

Tumor response was evaluated according to the Response Evaluation Criteria in Solid Tumors, version 1.1 (RECIST v1.1) [12] based on computed tomography (CT) findings. In addition, Tumor response was also evaluated according to the 12th edition of Japanese Classification of Esophageal Cancer (JES 12th) which described that the method of assessing the esophageal main lesion by CT with endoscopy after chemotherapy or radiotherapy [13]. The best overall response was assessed as complete response (CR), partial response (PR), stable disease (SD), non-CR/non-PD (only for RECIST v1.1), or progressive disease (PD). The overall response corresponded to the sum of the CR and PR, and disease control corresponded to the sum of the CR PR, SD, and non-CR/non-PD. The histopathological effects of chemotherapy were estimated according to JES 12th as follows: grade 0 (ineffective), grade 1a/1b (slightly effective), grade 2 (moderately effective), and grade 3 (markedly effective) [13].

All statistical analyses were performed using EZR (Saitama Medical Center, Jichi Medical University, Saitama, Japan), which is a graphical user interface for R (The R Foundation for Statistical Computing, Vienna, Austria); specifically, it is a modified version of R commander designed to add statistical functions that are frequently used in biostatistics [14].

## Results

### Patient characteristics

In total, 50 patients with ESCC were treated with preoperative DCF from July 2012 to December 2017 at The University of Tokyo Hospital. The cohort included 39 men and 11 women with a median age of 66 years (range 48–85). The median duration of observation was 26.7 months (range 3.9–104.6). Patients’ characteristics are presented in Table 1.

**Table 1:** Patient characteristics (n = 50)

Baseline characteristics	
Sex, n (%)	
Male	39 (78)
Age, years	
Median (range)	66 (48–85)
cStage, n (%)	
II	5 (10)
III	33 (66)
IV	12 (24)
Primary lesion location, n (%)	
Ce	4 (8)
Ut	3 (6)
Mt	29 (58)
Lt	10 (20)
Jz	4 (8)

*Ce* cervical esophagus, *Mt* middle thoracic esophagus, *Lt* lower thoracic esophagus, *Ut* upper thoracic esophagus, *Jz* zone of esophagogastric junction

### Treatment

Treatment-related death was not observed. Forty-seven patients completed both courses of preoperative DCF therapy. Three patients terminated treatment after one course because of toxicities. Of the 47 patients who completed treatment, the mean relative dose intensities (RDIs) of docetaxel, cisplatin, and 5-FU were 90.6% (range 75.7–100%), 88.0% (75.7–100%), and 90.6% (75.7–100%), respectively, and the mean average relative dose intensity (ARDI) during the two courses of DCF therapy was 89.7% (72.9–100%). One patient received one additional course of DCF therapy (three courses in total) because surgery was postponed treating coronary artery disease.

### Effectiveness

As presented in Table 2, the objective response rate was 30%/44%, and the disease control rate was 84%/84% by RECIST v1.1/JES 12th, respectively. Forty-four patients (88%) underwent surgery, including R0 (no residual tumor) resection in 42 patients and R2 (macroscopic residual tumor) resection in two patients. Their surgical strategy and surgical complications (by Clavien–Dingo classification [15]) are shown in Table 3. Among surgically treated patients, the histopathological effects of chemotherapy were as follows: grade 0, 0 (0%); grade 1, 33 (75%); grade 2, 9 (20%); grade 3, 1 (2%); and unknown, 1 (2%) (Table 4). Meanwhile, pathological lymph nodes were positive for metastasis (pN+) in 36 patients (82%) and negative for metastasis (pN0) in eight patients (18%), including two patients (5%) with pathological CR (pCR). Among the six patients who did not undergo surgery, five received concurrent chemoradiotherapy, and one received radiotherapy alone.

**Table 2:** Best overall response of neoadjuvant chemotherapy (n = 50)

Best overall response	RECIST v1.1	JES 12th
CR, n (%)	2 (4)	5 (10)
PR, n (%)	13 (26)	17 (34)
SD, n (%)	15 (30)	20 (40)
Non-CR/non-PD, n (%)	12 (24)	–
PD, n (%)	8 (16)	8 (16)
Objective response rate, %	30	44
Disease control rate, %	84	84

*RECIST v1.1* Response Evaluation Criteria in Solid Tumors, version 1.1, *JES 12th* Japanese Classification of Esophageal Cancer, 12th edition, *CR* complete response, *PR* partial response, *SD* stable disease, *PD* progressive disease

**Table 3:** Surgical strategy and complications (n = 44)

Characteristics	n (%)
Surgical approach	
Open esophagectomy	39 (89)
Thoracoscopic esophagectomy	0 (0)
Trans-mediastinal esophagectomy	3 (7)
TPLE	1 (2)
Number of lymph node dissection	
Median (range)	62 (0–155)
Residual tumor	
R0 (No residual tumor)	42 (95)
R1 (Microscopic residual tumor)	0 (0)
R2 (Macroscopic residual tumor)	2 (5)
Surgical complications	
None	17 (39)
Grade I	1 (2)
Grade II	15 (34)
Grade IIIa	5 (11)
Grade IIIb	1 (2)
Grade IVa	3 (7)
Grade V	2 (5)

*TPLE* total pharyngolaryngoesophagectomy,

**Table 4:** Histopathological effects of chemotherapy (n = 44)

Grade	n (%)
0	0 (0)
1a	24 (55)
1b	9 (20)
2	9 (20)
3	1 (2)
Not evaluable	1 (2)

**Fig. 1:**
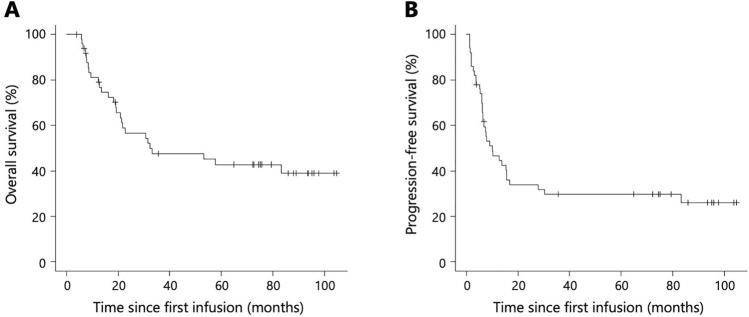
Kaplan–Meier curves of all patients treated with neoadjuvant DCF (n = 50). Median OS (**a**) and PFS (**b**) in the entire cohort

**Fig. 2:**
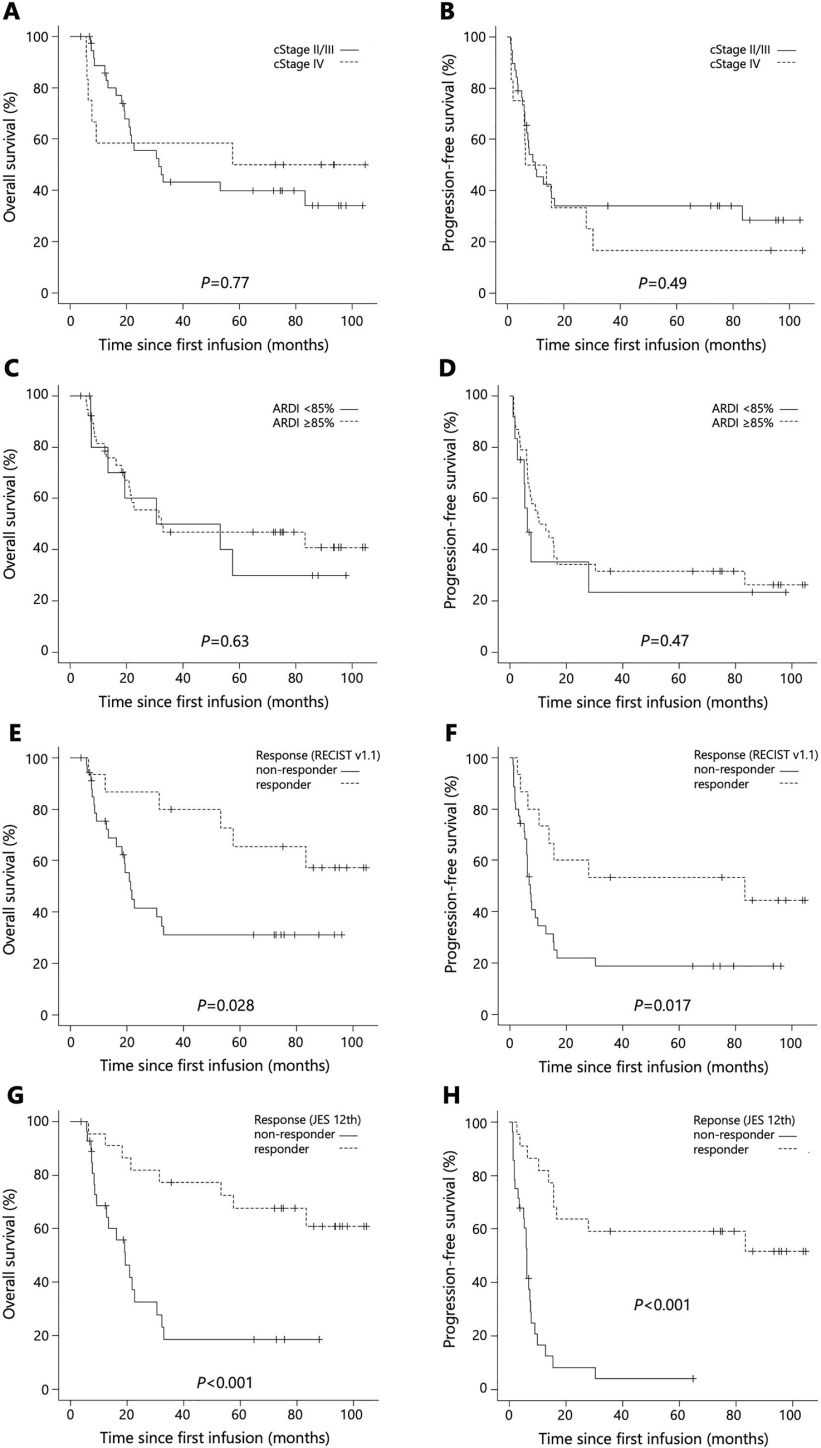
Stratified Kaplan–Meier curves of all patients treated with neoadjuvant DCF (n = 50). Median OS (**a**) and PFS (**b**) stratified by clinical stage (cStage I/III, n = 38; cStage IV, n = 12). Median OS (**c**) and PFS (**d**) stratified by ARDI (≥ 85%, n = 38; < 85%, n = 12). Median OS (**e**) and PFS (**f**) stratified by the objective response according to RECIST v1.1 (responders, n = 15; non-responders, n = 35) and median OS (**g**) and PFS (**h**) stratified by the objective response according to JES 12th (responders, n = 22; non-responders, n = 28). *OS* overall survival, *PFS* progression-free survival, *ARDI* average relative dose intensity, *RECIST v1.1* Response Evaluation Criteria in Solid Tumors, version 1.1, *JES 12th* Japanese Classification of Esophageal Cancer, 12th edition

Median OS and PFS in the entire cohort were 32.3 (95% CI 21.0–NA) and 10.0 months (95% CI 6.3–15.6), respectively (Fig. 1a, b). Stratified by clinical stage (cStage II/III, n = 38; cStage IV, n = 12), median OS was 31.5 months (95% CI 19.33–NA) in the cStage II/III subgroup, versus 57.6 months (95% CI 6.1–NA] in the cStage IV subgroup (p = 0.77; Fig. 2a). Median PFS was 10.0 months (95% CI 6.3–16.8) in the cStage II/III subgroup, versus 10.1 months (95% CI 1.4–30.3) in the cStage IV subgroup (p = 0.49; Fig. 2b). Stratified by ARDI (≥ 85%, n = 38; < 85%, n = 12), median OS was 32.3 months (95% CI 19.1–NA) in the high ARDI subgroup and 41.9 months (95% CI 7.4–NA) in the low ARDI subgroup (p = 0.63; Fig. 2c). Median PFS was 11.6 months (95% CI 6.4-NA] in the high ARDI subgroup and 6.3 months (95% CI 1.8–NA) in the low ARDI subgroup (p = 0.47; Fig. 2d). Stratified by the objective response according to RECIST v1.1 (responders, n = 15; non-responders, n = 35), median OS was not reached (95% CI 31.5–NA) in responders, versus 21.4 months (95% CI 13.5–33.0) in non-responders (p = 0.028; Fig. 2e). Median PFS was 83.3 months (95% CI 6.4–NA] in responders, versus 7.4 months (95% CI 6.0–12.8) in non-responders (p = 0.017; Fig. 2f). Stratified by the objective response according to JES 12th (responders, n = 22; non-responders, n = 28), median OS was not reached (95% CI 53.1–NA) in responders, versus 19.3 months (95% CI 9.4–30.6) in non-responders (p < 0.001; Fig. 2g). Median PFS was not reached (95% CI 15.6–NA] in responders, versus 6.2 months (95% CI 3.7–7.6) in non-responders (p < 0.001 Fig. 2h).

**Fig. 3:**
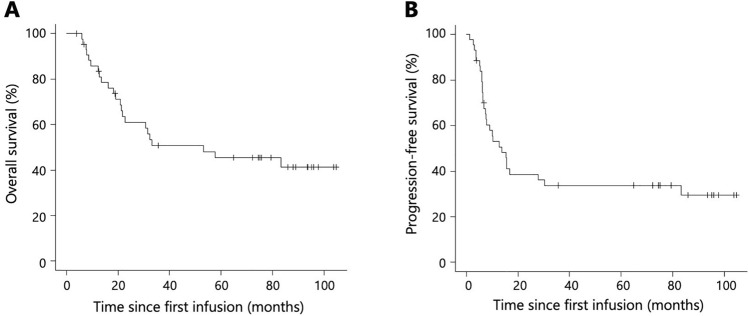
Kaplan–Meier curves of patients who underwent surgery after neoadjuvant DCF (n = 44). Median OS (**a**) and PFS (**b**)

**Fig. 4:**
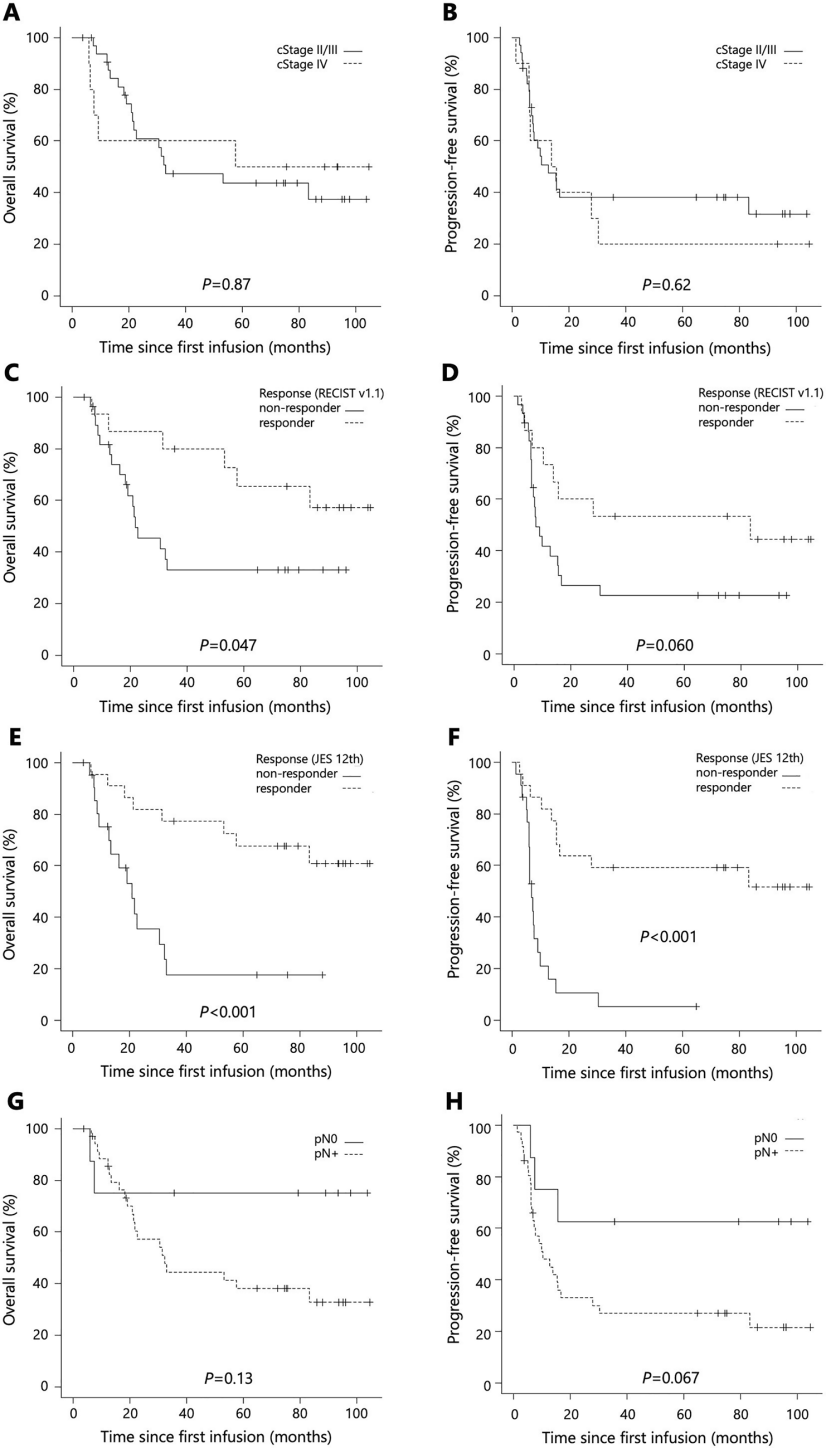
Kaplan–Meier curves of patients who underwent surgery after neoadjuvant DCF (n = 44). Median OS (**a**) and PFS (**b**) stratified by clinical stage (cStage II/III, n = 34; cStage IV, n = 10). Median OS (**c**) and PFS (**d**) stratified by the objective response according to RECIST v1.1 (responders, n = 15; non-responders, n = 29) and median OS (**e**) and PFS (**f**) stratified by the objective response according to JES 12th (responders, n = 22; non-responders, n = 22). Median OS (**g**) and PFS (**h**) stratified by the pathological lymph nodes metastasis status (pN0, n = 8; pN+, n = 36). *OS* overall survival, *PFS* progression-free survival, *ARDI* average relative dose intensity, *RECIST v1.1* Response Evaluation Criteria in Solid Tumors, version 1.1, *JES 12th* Japanese Classification of Esophageal Cancer, 12th edition, *pN0* pathological lymph nodes were negative for metastasis, pN+ pathological lymph nodes were positive for metastasis

Of the 44 surgically treated patients, median OS and PFS were 53.1 (95% CI 21.4–NA) and 13.8 months (95% CI 7.4–30.3), respectively (Fig. 3a, b). Stratified by clinical stage (cStage II/III, n = 34; cStage IV, n = 10), median OS was 33.1 months (95% CI 21.4–NA) in the cStage II/III subgroup and 57.6 months (95% CI 6.1–NA) in the cStage IV subgroup (p = 0.87; Fig. 4a). Median PFS was 12.8 months (95% CI 7.4–83.3) in the cStage II/III subgroup and 14.7 months (95% CI 1.4–30.3) in the cStage IV subgroup (p = 0.62; Fig. 4b). Stratified by the objective response according to RECIST v1.1 (responders, n = 15; non-responders, n = 29), median OS was not reached (95% CI 31.5–NA) in responders, versus 21.7 months (95% CI 16.2–NA) in non-responders (p = 0.047; Fig. 4c). Median PFS was 83.3 months (95% CI 6.4–NA) in responders, versus 7.8 months (95% CI 6.2–15.6) in non-responders (p = 0.060; Fig. 4d). Stratified by the objective response according to JES 12th (responders, n = 22; non-responders, n = 22), median OS was not reached (95% CI 53.1–NA) in responders, versus 21.0 months (95% CI 12.8–32.3) in non-responders (p < 0.001; Fig. 4e). Median PFS was not reached (95% CI 15.6–NA] in responders, versus 6.9 months (95% CI 6.0–9.1) in non-responders (p < 0.001 Fig. 4f). Stratified by the pathological lymph nodes metastasis status (pN0, n = 8; pN+, n = 36), median OS was not reached (95% CI 6.1–NA) in the pN0 subgroup and 32.3 months (95% CI 21.0–NA) in the pN+ subgroup (p = 0.13; Fig. 4g). Median PFS was not reached (95% CI 15.4–NA) in the pN0 subgroup, versus 10.3 months (95% CI 6.4–16.8) in the pN+ subgroup (p = 0.067; Fig. 4h).

## Discussion

In this study, we investigated the efficacy of neoadjuvant DCF chemotherapy followed by surgery for locally advanced esophageal cancer. Notably, we found that patients who responded to neoadjuvant DCF therapy had a good prognosis (both OS and PFS), and patients with pN+ were more likely to experience relapse (poor PFS). However, prognosis did not differ according to ARDI.

Our study revealed significantly longer OS and PFS in patients who responded to neoadjuvant DCF therapy. After excluding the six patients who did not proceed to surgery because of PD after preoperative DCF therapy, OS was significantly longer in responders, and the same trend was observed for PFS. However, outcomes did not differ between cStage IV and cStage II/III. A multicenter phase II trial of locally advanced unresectable (clinical T4) esophageal cancer evaluating DCF therapy followed by conversion surgery (if cancer became resectable) or concurrent chemoradiotherapy (if not resectable) found that OS and PFS were significantly longer for patients who underwent R0 resection (n = 19) than for those who did not undergo R0 resection (3-year OS: 71.4% vs. 30.1%, 3-year PFS: 61.3% vs 25.0%) [16]. Our results are consistent with these findings in that the outcome of sequential local treatment was better in responders. Based on these results, depending on the response to neoadjuvant DCF therapy, it could be possible to select patients who should undergo surgery (even if it is more invasive) because of the expected improvement in survival, whereas another modality treatment should be considered in other patients.

Moreover, our results identified pN+ as a risk factor for recurrence (poor PFS), although a significant difference was not obtained because of the small study size. A subgroup analysis of the JCOG9204 study comparing postoperative FP therapy with surgery alone suggested a trend of poorer prognosis for pN1 cases than for pN0 cases in both groups [2]. These consistent results suggest that pN+ is a risk factor for recurrence after preoperative chemotherapy followed by surgery in esophageal cancer. We are conducting a phase II study of neoadjuvant DCF therapy followed by adjuvant S-1 monotherapy for pN+ patients with locally advanced esophageal cancer (jRCTs031180375).

In neoadjuvant chemotherapy for early-stage breast cancer, the US Food and Drug Administration and European Medicines Agency have supported the use of pCR as a surrogate endpoint for long-term clinical benefit (event-free or disease-free survival and OS) for accelerated approval of new drugs in randomized clinical trials. Some previous meta-analyses reported a strong correlation between pCR and disease-free survival and OS, however, not at the trial level, but at the patient level (especially in aggressive tumor subtypes) [17, 18]. Our retrospective study for esophageal cancer showed consistent results that the effect of neoadjuvant chemotherapy could predict long-term clinical benefit, although the cancer type was different. Moreover, the study was characterized by including more advanced cases.

In our study, survival did not differ according to ARDI (ARDI ≥ 85% is equivalent to a one-level dose reduction for all three drugs). Although it is believed that the dose of perioperative chemotherapy as part of radical treatment cannot be easily reduced, in preoperative DCF therapy given every 4 weeks (two cycles) in esophageal cancer, dose reductions might be acceptable depending on adverse events and other patient factors. Conversely, in our ongoing phase II trial of cStage II/III esophageal cancer (jRCTs031180375), neoadjuvant DCF therapy is being administered every 3 weeks (three cycles) similarly as performed in the JCOG1109 trial [10]. Although the expected proportion of pN+ patients was expected to exceed 70% in jRCTs031180375 (versus 82% in the current study), when case accumulation was almost completed, the proportion of pN+ cases was much lower at 37%. Therefore, additional cases were added. These results suggest that adjuvant DCF therapy might be more effective if given every 3 weeks for three cycles than every 4 weeks for two cycles, although dose reduction is acceptable. However, a recent report of a multicenter randomized trial of two versus three courses of preoperative DCF therapy in 180 patients with locally advanced esophageal cancer, recorded comparable pN0 rates in the two groups (p = 0.225), with comparable R0 resection rates (98.9% vs 96.5%, p = 0.830) and 2-year PFS rate (71.4% vs. 71.1%, p = 0.669) [19]. Therefore, the number of courses of DCF administered might not affect its efficacy. Further studies are warranted to clarify the optimal dose, intervals, and course of neoadjuvant DCF therapy for esophageal cancer.

Several limitations of this study should be acknowledged. First, this was a retrospective study with a small number of patients, and selection bias might have arisen from physicians’ subjectivity in determining the application of adjuvant DCF therapy. Second, because the small cohort with 27 events of death was not statistically valid to perform a multivariate analysis with more than three variables, we could not conduct a multivariate analysis to assess the prognostic factors (including response to the DCF therapy or clinical staging) for survivals.

In conclusion, the present study found that neoadjuvant DCF chemotherapy followed by surgery for locally advanced esophageal cancer was feasible, and the response to neoadjuvant DCF therapy could predict the long-term prognosis of patients. Additionally, pN + tended to increase the risk of recurrence, but cStage (II/III or IV) and lower ARDI did not influence survival.

## Data Availability

The data supporting the findings of this study are available from the corresponding author upon reasonable request.

## References

[1] Cancer Information Service. Cancer Statistics in Japan 2021 [Internet]. Foundation for Promotion of Cancer Research; 2021. http://ganjoho.jp/en/professional/statistics/brochure/2013_en.html.

[2] Ando N, Iizuka T, Ide H, Ishida K, Shinoda M, Nishimaki T, et al. Surgery plus chemotherapy compared with surgery alone for localized squamous cell carcinoma of the thoracic esophagus: a Japan Clinical Oncology Group Study—JCOG9204. J Clin Oncol. 2003;21:4592–6. 14673047 10.1200/JCO.2003.12.095

[3] Ando N, Kato H, Igaki H, Shinoda M, Ozawa S, Shimizu H, et al. A randomized trial comparing postoperative adjuvant chemotherapy with cisplatin and 5-fluorouracil versus preoperative chemotherapy for localized advanced squamous cell carcinoma of the thoracic esophagus (JCOG9907). Ann Surg Oncol. 2012;19:68–74. 21879261 10.1245/s10434-011-2049-9

[4] Van Cutsem E, Moiseyenko VM, Tjulandin S, Majlis A, Constenla M, Boni C, et al. Phase III study of docetaxel and cisplatin plus fluorouracil compared with cisplatin and fluorouracil as first-line therapy for advanced gastric cancer: a report of the V325 study group. J Clin Oncol [Internet]. 2006;24:4991–7. 10.1200/JCO.2006.06.8429. 17075117 10.1200/JCO.2006.06.8429

[5] Vermorken JB, Remenar E, van Herpen C, Gorlia T, Mesia R, Degardin M, et al. Cisplatin, fluorouracil, and docetaxel in unresectable head and neck cancer. N Engl J Med [Internet]. 2007;357:1695–704. 10.1056/NEJMoa071028. 17960012 10.1056/NEJMoa071028

[6] Posner M, Hershock D. Cisplatin and fluorouracil alone or with docetaxel in head and neck cancer. N Engl J Med [Internet]. 2007. 10.1056/NEJMoa070956. 17960013 10.1056/NEJMoa070956

[7] Chiarion-Sileni V, Corti L, Ruol A, Innocente R, Boso C, Del Bianco P, et al. Phase II trial of docetaxel, cisplatin and fluorouracil followed by carboplatin and radiotherapy in locally advanced oesophageal cancer. Br J Cancer. 2007;96:432–8. 17245338 10.1038/sj.bjc.6603585 PMC2360020

[8] Hara H, Tahara M, Daiko H, Kato K, Igaki H, Kadowaki S, et al. Phase II feasibility study of preoperative chemotherapy with docetaxel, cisplatin, and fluorouracil for esophageal squamous cell carcinoma. Cancer Sci. 2013;104:1455–60. 23991649 10.1111/cas.12274 PMC7654256

[9] Yamasaki M, Yasuda T, Yano M, Hirao M, Kobayashi K, Fujitani K, et al. Multicenter randomized phase II study of cisplatin and fluorouracil plus docetaxel (DCF) compared with cisplatin and fluorouracil plus Adriamycin (ACF) as preoperative chemotherapy for resectable esophageal squamous cell carcinoma (OGSG1003). Ann Oncol [Internet]. 2017;28:116–20. 10.1093/annonc/mdw439. 27687307 10.1093/annonc/mdw439

[10] Kato K, Ito Y, Daiko H, Ozawa S, Ogata T, Hara H, et al. A randomized controlled phase III trial comparing two chemotherapy regimen and chemoradiotherapy regimen as neoadjuvant treatment for locally advanced esophageal cancer, JCOG1109 NExT study. J Clin Oncol [Internet]. 2022;40:238. 10.1200/JCO.2022.40.4_suppl.238. 10.1200/JCO.2022.40.4_suppl.238

[11] Sobin LH, Gospodarowicz MK, Wittekind C. TNM classification of malignant tumours. 7th ed. New York: Wiley-Blackwell; 2011.

[12] Eisenhauer EA, Therasse P, Bogaerts J, Schwartz LH, Sargent D, Ford R, et al. New response evaluation criteria in solid tumours: Revised RECIST guideline (version 1.1). Eur J Cancer. 2009;45:228–47. 19097774 10.1016/j.ejca.2008.10.026

[13] Doki Y, Tanaka K, Kawachi H, Shirakawa Y, Kitagawa Y, Toh Y, et al. Japanese classification of esophageal cancer, 12th edition: part II [Internet]. Esophagus. 2024. 10.1007/s10388-024-01048-w. 38568243 10.1007/s10388-024-01048-w PMC11199297

[14] Kanda Y. Investigation of the freely available easy-to-use software “EZR” for medical statistics. Bone Marrow Transplant. 2013;48:452–8. 23208313 10.1038/bmt.2012.244 PMC3590441

[15] Dindo D, Demartines N, Clavien PA. Classification of surgical complications: a new proposal with evaluation in a cohort of 6336 patients and results of a survey. Ann Surg. 2004;240:205–13. 15273542 10.1097/01.sla.0000133083.54934.ae PMC1360123

[16] Yokota T, Kato K, Hamamoto Y, Tsubosa Y, Ogawa H, Ito Y, et al. A 3-year overall survival update from a phase 2 study of chemoselection with DCF and subsequent conversion surgery for locally advanced unresectable esophageal cancer. Ann Surg Oncol [Internet]. 2020;27:460–7. 10.1245/s10434-019-07654-8. 31376034 10.1245/s10434-019-07654-8

[17] Cortazar P, Zhang L, Untch M, Mehta K, Costantino JP, Wolmark N, et al. Pathological complete response and long-term clinical benefit in breast cancer: the CTNeoBC pooled analysis. Lancet. 2014;384:164–72. 24529560 10.1016/S0140-6736(13)62422-8

[18] Conforti F, Pala L, Sala I, Oriecuia C, De Pas T, Specchia C, et al. Evaluation of pathological complete response as surrogate endpoint in neoadjuvant randomised clinical trials of early stage breast cancer: systematic review and meta-analysis. BMJ. 2021;375:1–13. 10.1136/bmj-2021-066381 PMC8689398 34933868

[19] Makino T, Yamasaki M, Tanaka K, Yamashita K, Urakawa S, Ishida T, et al. Multicenter randomised trial of two versus three courses of preoperative cisplatin and fluorouracil plus docetaxel for locally advanced oesophageal squamous cell carcinoma. Br J Cancer. 2022. 10.1038/s41416-022-01726-5 PMC9130290 35140339

